# Sex-related differences in endothelial function and blood viscosity in the elderly population

**DOI:** 10.3389/fphys.2023.1151088

**Published:** 2023-03-30

**Authors:** Antoine Raberin, Cyril Martin, Sébastien Celle, David Hupin, Frederic Roche, Jean-Claude Barthelemy, Philippe Connes

**Affiliations:** ^1^ Institute of Sport Sciences, Université de Lausanne, Lausanne, Switzerland; ^2^ Inter-University Laboratory of Human Movement Biology, University Claude Bernard Lyon 1, University of Lyon, Lyon, France; ^3^ Service de Physiologie Clinique et de l’Exercice, CHU Saint Etienne, Saint-Etienne, France; ^4^ Jean Monnet University Saint-Etienne, Mines Saint-Etienne, University Hospital of Saint-Etienne, INSERM, U1059, DVH Team, SAINBIOSE, Saint-Etienne, France

**Keywords:** cardiovascular health, aging, cardiovascular disease, sex effect, hemorheology

## Abstract

Elderly represents a growing population and cardiovascular diseases (CVD) is one of the leading causes of mortality in this population. Sex differences are involved in CVD with middle-aged males being at higher risk than females. After menopause, females are no longer protected by hormones and the role of sex on cardiovascular parameters involved in CVD, such as endothelial function and blood viscosity, is still unclear. The purpose of this study was to investigate the effect of sex on endothelial function, blood viscosity and CVD in elderly. Clinical investigation and blood analyses were performed on 182 (93 females and 89 males) elderly participants (mean age: 75.83 ± 1.22). Health status of participants were classified. Sex differences in endothelial function, blood viscosity, high density lipoprotein (HDL), hematocrit, and red blood cell (RBC) aggregation were assessed. CVD prevalence was higher in males (27.0%) than in females (5.4%) (*p* < 0.001). Females had higher vasoreactivity (*p* = 0.014) and HDL (*p* < 0.001) level than males. Blood viscosity was higher in males than in females at any shear rate (*p* < 0.001). Hematocrit was greater in males than in females (*p* < 0.001) while RBC aggregation did not differ between the two populations. To conclude, females have less CVD than age-matched males that might be due to their greater vascular function and lower blood viscosity.

## 1 Introduction

Elderly represents a growing population in industrialized country particularly exposed to cardiovascular diseases (CVD) which remain the leading cause of death in western societies (Alexander et al., 2007). The risk of CVD, such as atherothrombotic disease, increases over the years (Alexander et al., 2007). This association between the increased risk of CVD and the rise in life expectancy led to challenges for public health and cardiovascular medicine. Although the elderly population continuously increases among patients (Alexander et al., 2007), elderly (>65 years) patients are under-represented in clinical trials. Learning societies report that only 10% of clinical trials’ participant are aged over 75 years old (Alexander et al., 2007). As a result, elderly population is more exposed to cardiovascular risk factors and less likely to receive the most efficient care and support. In this context the longitudinal cohort study “PROgnostic indicator OF cardiovascular and cerebrovascular events” (PROOF) has been conducted (Barthélémy et al., 2007; Raberin et al., 2020a) (1,011 elderly subjects, mean age upon study inclusion: 65.64 ± 0.8). Previous results on this cohort after a 10-years follow-up (mean age: 75.80 ± 1.2) reported an independent sex effect on several cardiovascular risk high density lipoprotein factors such as oxidative stress markers and high density lipoprotein (HDL) level (Raberin et al., 2020a). In this population of 75 years old, females had less cardiovascular risks and were less affected by CVD. This result highlight that even though women were menopaused and less protected by the effects estrogen (Novella et al., 2012), they are still more protected than men long time after menopause ended. Young and middle aged women are known to benefit from the protective role of estrogen on cardiovascular system (Merz and Cheng, 2016). However, the loss of hormone protective effect at menopause makes them more at risk after this period (Nappi et al., 2022). Hence, stiffer arteries in postmenopausal women have been reported compared to men (Mitchell et al., 2008). The exact benefit of previous exposure to estrogen remains unclear with an estimated loss of hormone-related cardio protection around 10 years after menopause (Nappi et al., 2022). In this context, the lower CVD and cardiovascular risk factors at 75.80 years old in women (Raberin et al., 2020a) suggested that benefit from previous long-term exposure to estrogen or sex-related cardiovascular protection still exist.

CVD are commonly associated with increased arterial stiffness and/or impaired vascular function. However, hemorheological properties also play a key role in the development and progression of vascular disorders and dysfunction (Cho et al., 2014). Both, blood viscosity and aging are associated with delayed or blunted flow-mediated dilation in middle age individuals (mean age 58.4 years) (Irace et al., 2015). Although aging is characterized by a decrease in hematocrit (Hct) after 80 years, a rise in blood viscosity was also observed and mainly due to increased RBC aggregation and to a lower extent to the rise in plasma viscosity (Raberin et al., 2022). Unfortunately, although playing a determinant role in blood perfusion, blood viscosity properties are scarcely investigated in the context of CVD (Baskurt and Meiselman, 2003; Celik et al., 2016) and more particularly in elderly population. Apart the already described lower Hct in women (Gudmundsson and Bjelle, 1993), sex-related difference in blood viscosity and its determinants remain to be fully characterized. One previous study reported lower blood viscosity, RBC deformability and Hct in obese middle-aged women compared to matched men (Wiewióra et al., 2010). Whether blood viscosity and vascular function could be better preserved in post-menopausal females than in males of 75 years old and participate to the lower prevalence of CVD is unknown.

The aim of the present study was to compare vascular function, blood viscosity and the prevalence of cardiovascular diseases in a population aged of 75 years old who belonged to the PROOF cohort (Raberin et al., 2020a). We hypothesized that elderly males would have lower vascular reactivity, higher blood viscosity, and would exhibit a higher prevalence of CVD compared to females.

## 2 Materials and methods

### 2.1 Protocol

The present study was part of the PROOF study which originally included 1,011 elderly subjects from the electoral list of the city of Saint-Etienne (France) aged of 65 years old upon study inclusion. Subjects with previous cardiovascular events, Parkinson’s disease, type 1 diabetes, or with life-expectancy of less than 5 years, and those who were dependent or living in a retirement home were excluded from the study (Barthélémy et al., 2007). The PROOF study was approved by the Ethics Committee (CCPRB, Loire, France), the National Committee for Information and Liberty (CNIL) gave its consent for data collection (NCT 00759304), and all subjects gave their written informed consent.

A follow-up was implemented every 2 years for 10 years. A new collection of clinical complications and biomarkers was done in 2010–2011 on volunteers (mean age: 75.83 ± 1.22) (Raberin et al., 2020a). Hemorheological properties and endothelial function measurements were performed on a subset of 182 subjects (93 females, 89 males).

### 2.2 Clinical examination and vascular function

During each clinical evaluation, blood was sampled from the antecubital vein after 12 h of fasting. Then, medical histories, examination, and treatment were recorded. Missing information was received from hospital charts, reviews, and questionnaires sent to the practitioners of the family. Health status of participants were classified by physicians among healthy, cardiovascular, cancer, or neurodegenerative disease.

**FIGURE 1:**
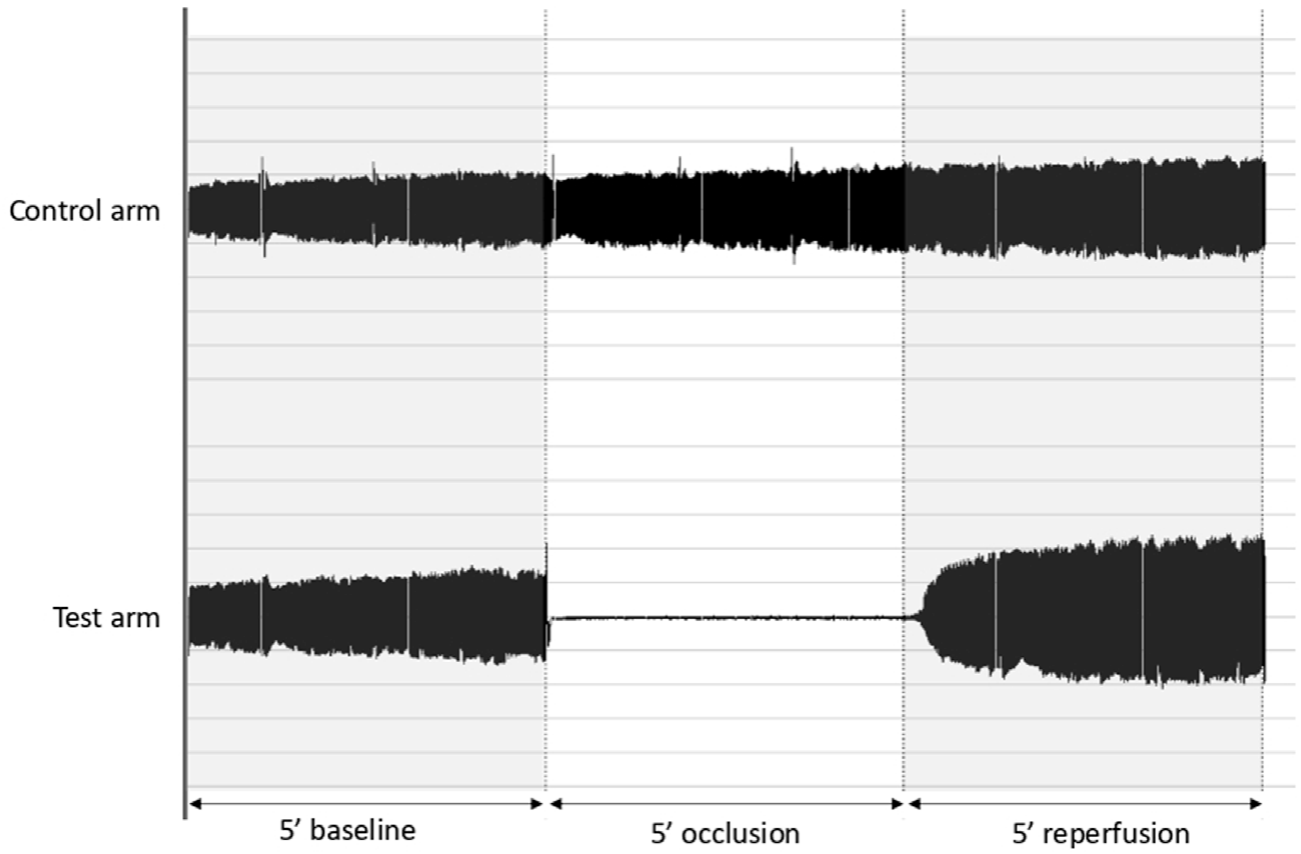
Typical signals obtain during the vascular function evaluation. After 5 min baseline measurement, a blood pressure cuff on the test arm was inflated for 5 min. After 5 min occlusion, the cuff was deflated, and the signal recorded for 5 min of reperfusion.

Vascular function was assessed during the clinical examination by vascular reactivity using digital tonometry (EndoPAT, Itamar Medical, Atlanta, United States) (Hansen et al., 2017). This technic allows the calculation of the reactive hyperemia index (RHI) from peripheral arterial tonometry, which assessed digital volume changes occurring with pulse waves. Briefly, probes comprising a system of inflatable latex air cuffs connected by pneumatic tubes to an inflating device were placed on the middle finger of participants’ hand. Pulsatile volume changes of the distal digit induced pressure alterations in the finger cuff, which were sensed by pressure transducers (Hamburg and Benjamin, 2009). A reactive hyperemic protocol was applied as follow. After 5 min baseline measurement, a blood pressure cuff on the test arm was inflated to 80 mmHg above baseline systolic blood pressure and at least 200 mmHg for 5 min. After 5 min occlusion, the cuff was deflated, and the signal recorded for 5 min. The ratio of the peripheral arterial tonometry signal after cuff release compared with baseline was calculated through a computer algorithm automatically normalizing for baseline signal and indexed to the contra lateral arm (Figure 1). The calculated ratio reflects the RHI.

### 2.3 Blood samples analysis

Cholesterol enzymatic method was used to measure HDL after a selective immune separation in homogenous phase (Cobas Integra 400+ analyzer, Roche Diagnostics Gmbh, Mannheim, Germany) (Bachorik et al., 1988).

Hematocrit (Hct) level was determined by microcentrifugation of glass capillaries filled with blood (Baskurt et al., 2009). Blood viscosity was determined after complete blood oxygenation, at native hematocrit and different shear rates (22.5, 45, 90 and 225 s^−1^) using a cone-plate viscometer (Brookfield DVII+ with CPE40 spindle, Brookfield Engineering Labs, Natick, MA). Red blood cell (RBC) aggregation was measured by light transmission with the Myrenne aggregometer (Schmid-Schonbein et al., 1990). Before measurement, the suspension was sheared at 600 s^−1^ to dissociate pre-existing aggregates and Hct of the suspension was standardized to 40% to avoid any influence of the concentration of red blood cells on RBC aggregation. After pre-existing RBC aggregates were dissociated, shearing was either stop or decreased to 10 s^−1^ in order to determine two indices of RBC aggregation: M index: RBC aggregation at stasis; M1 index: RBC aggregation at low shear rate. Hemorheological analyses were performed within 30 min after blood sampling to avoid any blood alteration (Baskurt et al., 2009).

### 2.4 Statistical analysis

All statistical analyses were performed using SPSS software (Chicago, IL, United States). Chi^2^ test was used to analyze the distribution of medical status. Quantitative data were compared using a Student *t*-test or a signed-rank test of Mann-Whitney according to the normality of the distribution of the data, which was assessed by a Shapiro-Wilk test. A *p*-value < 0.05 was considered statistically significant.

## 3 Results

The repartition of males and females in each medical status was significantly different [X^2^(3) = 18.15, *p* < 0.001, Table 1]. CVD were the most frequent diseases in the cohort and CVD frequency was 5 times higher in males than in females (*p* < 0.001).

**TABLE 1:** Repartition of males and females in the medical status.

	Healthy	Cancer	CVD	Neurodegenerative
Females (93)	86.0% (80)	5.4% (5)	5.4% (5)	3.2% (3)
Males (89)	69.7% (62)	3.4% (3)	27.0% (24)	0.0% (0)

CVD, cardiovascular diseases; X^2^ (3) = 18.15, *p* < 0.001.

**FIGURE 2:**
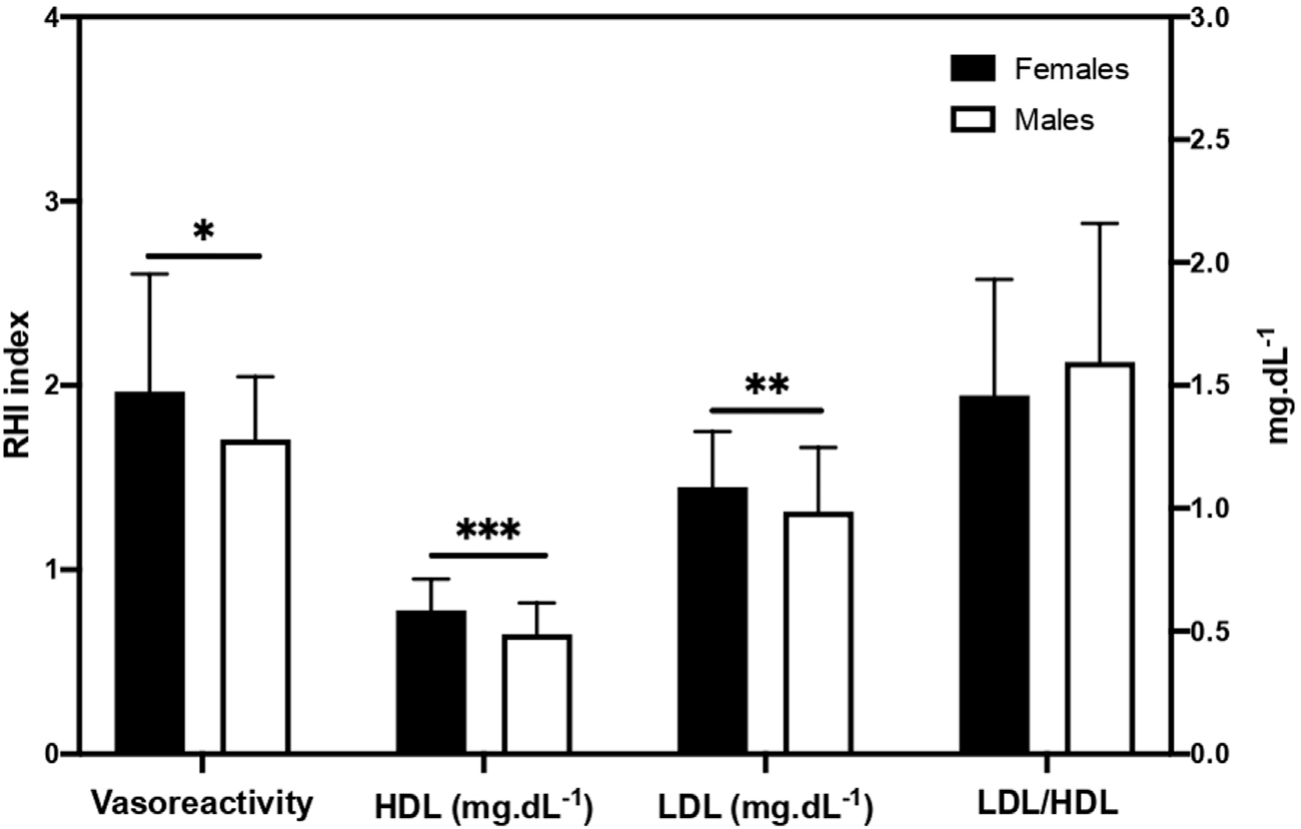
Reactive hyperemia index and lipoproteins. Data are mean ± SD. HDL, high density lipoprotein; LDL, low-density lipoprotein; RHI, reactive hyperemia index. LDL/HDL ratio values refer to the right axis. Significantly different between males and females: *p < 0.05, **p < 0.01, ***p < 0.001.

Sex differences were observed concerning RHI and lipid biochemical parameters. Females had higher RHI (*p* = 0.014), HDL (*p* < 0.001) and LDL (*p* = 0.007) levels than males (Figure 2). The lower LDL/HDL ratio in females compared to males did not reach statistical significance (*p* = 0.078).

**FIGURE 3:**
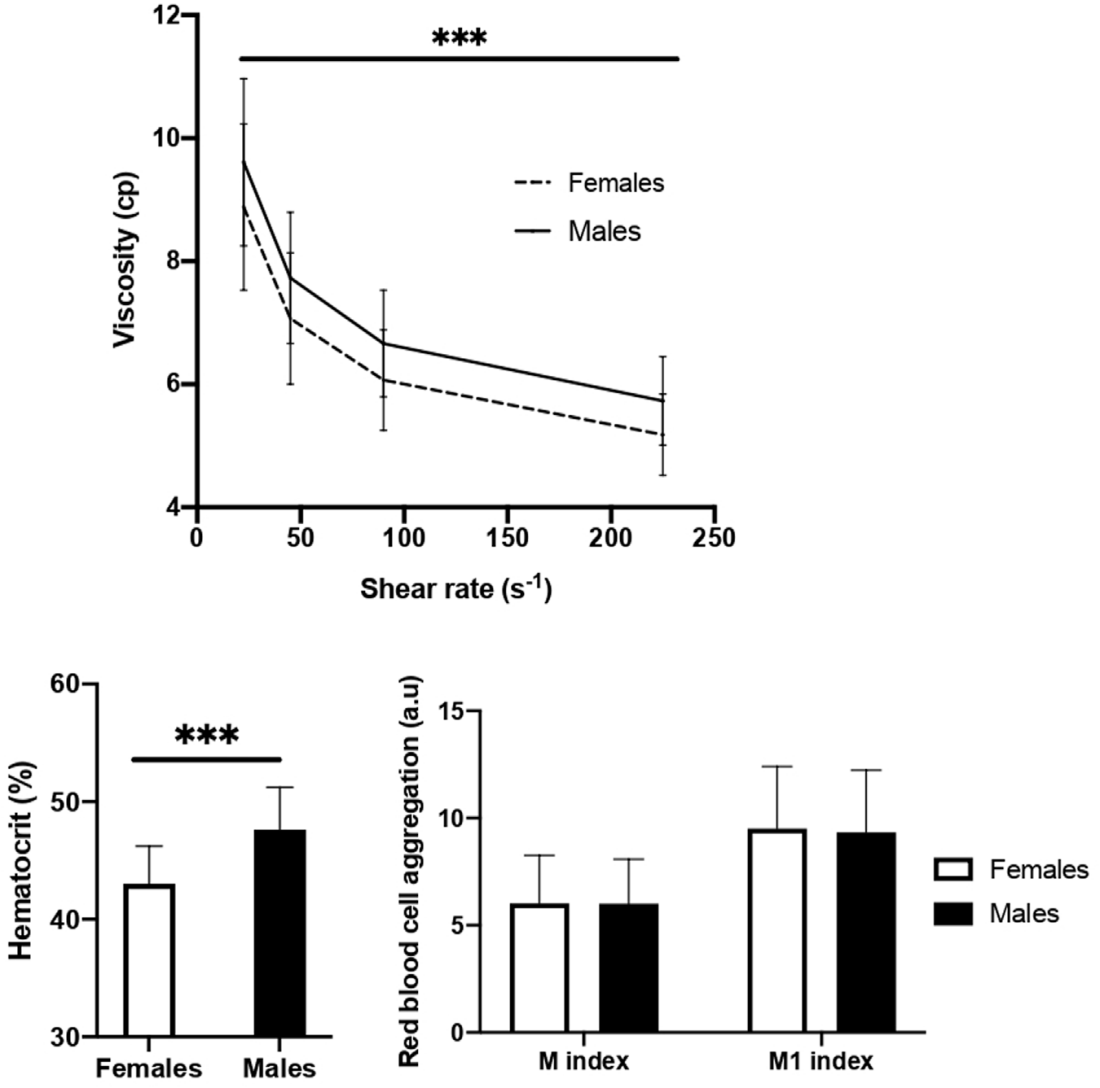
Blood viscosity and its determinants. Data are mean ± SD. M index: aggregation at stasis, M1 index: aggregation at low shear rate. Significantly different between males and females ***p < 0.001.

Blood viscosity was higher in males than in females at all shear rates (*p* < 0.001). Among determinants of blood viscosity, only Hct differed among sex, with a significantly higher Hct in males than in females (*p* < 0.001) (Figure 3).

## 4 Discussion/conclusion

The purpose of this study was to investigate sex-related differences in vascular function and blood viscosity and to compare the prevalence of cardiovascular diseases between elderly females and males.

Although the loss of sexual hormone cardioprotective effect in females after menopause should lead to an increased risk of CVD, females still had a lower prevalence of CVD than males (Raberin et al., 2020a). One could hypothesize that previous exposure to estrogen could have reduced vascular aging, and hence, that females would benefit from delayed vascular aging after menopause (Novella et al., 2012). A smaller age-related increase in arterial stiffening in females than in males was reported and ascribed to sex hormones differences (Astrand et al., 2011). Moreover, endothelial dependent vasodilation, measured by flow mediated dilation, has been shown to decline earlier in males than in females but a step decrease was reported at the time of menopause (Celermajer et al., 1994). Indeed, in the present study, females also exhibited higher vascular reactivity measured by reactive hyperemia than males. The greater RHI could be, at least partly, explained by the higher HDL and the trend toward lower LDL/HDL ratio in females. In addition to serving classic functions in cholesterol homeostasis and reverse cholesterol transport, it has been reported that HDL promote the production of NO (Besler et al., 2012). HDL have a protecting effect against alteration of endothelial NO synthase due to oxidized LDL (Besler et al., 2012). HDL also directly stimulate endothelial NO synthase by phosphorylation at serine residue-1177 which explain the impact of HDL on NO production (Besler et al., 2012). Hence, NO bioavailability could be higher in elderly females especially considering that it was previously reported in the full PROOF cohort that females had less oxidative stress and higher HDL than males (Raberin et al., 2020a).

Another explanation of the greater vascular health and lower prevalence of CVD in females could be attributed to the difference in blood flow properties, which may be involved in the expansion of vascular dysfunction (Cho et al., 2014). The present study reports that females had lower blood viscosity at high and low shear rates than males. This should have induced less stress on endothelial wall and greater blood perfusion. The higher blood viscosity found in males, in a context of a lower vascular reactivity, would lead to increased vascular resistance, promoting endothelial damages, increased blood pressure and decreased blood perfusion. This may increase the risk of a self-perpetuating cycle involving increased blood pressure and arterial stiffening ultimately leading to CVD. Moreover, decreased blood perfusion is also involved in the pathophysiology of many age-related diseases, such as brain diseases or coronary diseases. The lower blood viscosity in females may be attributed to the lower hematocrit (Raberin et al., 2022). Although a decline in Hct occurred during aging, it appears mainly in advanced age (80 years old) (Raberin et al., 2022). Males and females of the present study were unlikely to exhibit an aged-related decline in Hct with values being withing the normal population range (41%–51% and 36%–44% for males and females, respectively). Increased RBC aggregation may also disturb blood flow in both the macro- and microcirculation, and has been shown to increase the risk of atherothrombosis complications (Raberin et al., 2022). Previous studies in elderly showed that aging was characterized by a slight increase in RBC aggregation (Raberin et al., 2022). The increased fibrinogen concentration related to silent inflammation and/or the decrease in RBC membrane sialic acid occurring with aging may lead to higher RBC aggregation (Raberin et al., 2022). However, age-related increased in RBC aggregation was not observed in the present study, as elderly people values were comparable to younger population (Raberin et al., 2020b). Our results show that RBC aggregation level is not affected by sex in 75 years old individuals and suggest that it would not play a role in the greater CVD risk found in males. Unfortunately, RBC deformability, another determinant of blood viscosity, was not investigated during this study making it difficult to conclude on its role in the observed sex-related difference.

In conclusion, the better vascular function, greater HDL, and lower blood viscosity in elderly females could participate in decreasing the risk of CVD in females compared to age-matched males.

## Data Availability

The raw data supporting the conclusion of this article will be made available by the authors, without undue reservation.

## References

[B1] Alexander K. P. Newby L. K. Cannon C. P. Armstrong P. W. Gibler W. B. Rich M. W. (2007). Acute coronary care in the elderly, part I: Non-ST-segment-elevation acute coronary syndromes: A scientific statement for healthcare professionals from the American heart association council on clinical cardiology: In collaboration with the society of geriatric cardiology. Circulation 115, 2549–2569. 10.1161/CIRCULATIONAHA.107.182615 17502590

[B2] Astrand H. Stalhand J. Karlsson J. Karlsson M. Sonesson B. Länne T. (2011). *In vivo* estimation of the contribution of elastin and collagen to the mechanical properties in the human abdominal aorta: Effect of age and sex. J. Appl. Physiol. 110, 176–187. 10.1152/japplphysiol.00579.2010 21071586

[B3] Bachorik P. S. Virgil D. G. Derby C. Widman D. McMahon R. Fulwood R. P. (1988). Enzymatic analysis of total- and HDL-cholesterol: Comparison with the standardized liebermann-burchard method used by the lipid research clinics program. Clin. Chim. Acta 174, 307–314. 10.1016/0009-8981(88)90057-5 3390958

[B4] Barthélémy J.-C. Pichot V. Dauphinot V. Celle S. Laurent B. Garcin A. (2007). Autonomic nervous system activity and decline as prognostic indicators of cardiovascular and cerebrovascular events: The “PROOF” study. Study design and population sample. Associations with sleep-related breathing disorders: The “SYNAPSE” study. Neuroepidemiology 29, 18–28. 10.1159/000108914 17898520

[B5] Baskurt O. K. Boynard M. Cokelet G. C. Connes P. Cooke B. M. Forconi S. (2009). New guidelines for hemorheological laboratory techniques. Clin. Hemorheol. Microcirc. 42, 75–97. 10.3233/CH-2009-1202 19433882

[B6] Baskurt O. K. Meiselman H. J. (2003). Blood rheology and hemodynamics. Semin. Thromb. Hemost. 29, 435–450. 10.1055/s-2003-44551 14631543

[B7] Besler C. Lüscher T. F. Landmesser U. (2012). Molecular mechanisms of vascular effects of high-density lipoprotein: Alterations in cardiovascular disease. EMBO Mol. Med. 4, 251–268. 10.1002/emmm.201200224 22431312 PMC3376856

[B8] Celermajer D. S. Sorensen K. E. Spiegelhalter D. J. Georgakopoulos D. Robinson J. Deanfield J. E. (1994). Aging is associated with endothelial dysfunction in healthy men years before the age-related decline in women. J. Am. Coll. Cardiol. 24, 471–476. 10.1016/0735-1097(94)90305-0 8034885

[B9] Celik T. Balta S. Ozturk C. Iyisoy A. (2016). Whole blood viscosity and cardiovascular diseases: A forgotten old player of the game. Med. Princ. Pract. 25, 499–500. 10.1159/000446916 27194430 PMC5588435

[B10] Cho Y. I. Cho D. J. Rosenson R. S. (2014). Endothelial shear stress and blood viscosity in peripheral arterial disease. Curr. Atheroscler. Rep. 16, 404. 10.1007/s11883-014-0404-6 24519415

[B11] Gudmundsson M. Bjelle A. (1993). Plasma, serum and whole-blood viscosity variations with age, sex, and smoking habits. Angiology 44, 384–391. 10.1177/000331979304400507 8480916

[B12] Hamburg N. M. Benjamin E. J. (2009). Assessment of endothelial function using digital pulse amplitude tonometry. Trends Cardiovasc Med. 19, 6–11. 10.1016/j.tcm.2009.03.001 19467447 PMC3777618

[B13] Hansen A. S. Butt J. H. Holm-Yildiz S. Karlsson W. Kruuse C. (2017). Validation of repeated endothelial function measurements using EndoPAT in stroke. Front. Neurol. 8, 178. 10.3389/fneur.2017.00178 28515707 PMC5413501

[B14] Irace C. Tripolino C. Scavelli F. Messiniti V. Tassone B. Della Valle E. (2015). Blood viscosity but not shear stress associates with delayed flow-mediated dilation. Eur. J. Appl. Physiol. 115, 747–753. 10.1007/s00421-014-3058-8 25428725

[B15] Merz A. A. Cheng S. (2016). Sex differences in cardiovascular ageing. Heart 102, 825–831. 10.1136/heartjnl-2015-308769 26917537 PMC5993677

[B16] Mitchell G. F. Gudnason V. Launer L. J. Aspelund T. Harris T. B. (2008). Hemodynamics of increased pulse pressure in older women in the community-based Age, Gene/Environment Susceptibility-Reykjavik Study. Hypertension 51, 1123–1128. 10.1161/HYPERTENSIONAHA.107.108175 18259005 PMC11106724

[B17] Nappi R. E. Chedraui P. Lambrinoudaki I. Simoncini T. (2022). Menopause: A cardiometabolic transition. Lancet Diabetes Endocrinol. 10, 442–456. 10.1016/S2213-8587(22)00076-6 35525259

[B18] Novella S. Dantas A. P. Segarra G. Medina P. Hermenegildo C. (2012). Vascular aging in women: Is estrogen the fountain of youth? Front. Physiol. 3, 165. 10.3389/fphys.2012.00165 22685434 PMC3368545

[B19] Raberin A. Burtscher J. Connes P. Millet G. P. (2022). Hypoxia and hemorheological properties in older individuals. Ageing Res. Rev. 79, 101650. 10.1016/j.arr.2022.101650 35597435

[B20] Raberin A. Connes P. Barthélémy J.-C. Robert P. Celle S. Hupin D. (2020a). Role of gender and physical activity level on cardiovascular risk factors and biomarkers of oxidative stress in the elderly. Oxid. Med. Cell Longev. 2020, 1315471. 10.1155/2020/1315471 32655757 PMC7321518

[B21] Raberin A. Nader E. Ayerbe J. L. Mucci P. Connes P. Durand F. (2020b). Evolution of blood rheology and its relationship to pulmonary hemodynamic during the first days of exposure to moderate altitude. Clin. Hemorheol. Microcirc. 74, 201–208. 10.3233/CH-190671 31476150

[B22] Schmid-Schonbein H. Malotta H. Striesow F. (1990). Erythrocyte aggregation: Causes, consequences and methods of assessment. Tijdschr. NvKC 15, 88–97.

[B23] Wiewióra M. Sosada K. Slowinska L. Piecuch J. Glück M. Zurawinski W. (2010). Sex-dependent differences in rheological properties and the relation of blood viscosity to erythrocyte aggregation indices among morbidly obese patients. Clin. Hemorheol. Microcirc. 44, 259–267. 10.3233/CH-2010-1275 20571240

